# Improving the trajectory of transpedicular transdiscal lumbar screw fixation with a computer-assisted 3D-printed custom drill guide

**DOI:** 10.7717/peerj.3564

**Published:** 2017-07-13

**Authors:** Zhen-Xuan Shao, Jian-Shun Wang, Zhong-Ke Lin, Wen-Fei Ni, Xiang-Yang Wang, Ai-Min Wu

**Affiliations:** Department of Spine Surgery, The Second Affiliated Hospital and Yuying Children’s Hospital of Wenzhou Medical University, Second Medical School of Wenzhou Medical University, Digital Orthopedic Institute, Zhejiang Spine Surgery Center, Wenzhou, Zhejiang, China

**Keywords:** Transpedicular transdiscal lumbar screw fixation, Three dimensional printed template, Lumbar spine, Three dimensional reconstruction

## Abstract

Transpedicular transdiscal screw fixation is an alternative technique used in lumbar spine fixation; however, it requires an accurate screw trajectory. The aim of this study is to design a novel 3D-printed custom drill guide and investigate its accuracy to guide the trajectory of transpedicular transdiscal (TPTD) lumbar screw fixation. Dicom images of thirty lumbar functional segment units (FSU, two segments) of L1–L4 were acquired from the PACS system in our hospital (patients who underwent a CT scan for other abdomen diseases and had normal spine anatomy) and imported into reverse design software for three-dimensional reconstructions. Images were used to print the 3D lumbar models and were imported into CAD software to design an optimal TPTD screw trajectory and a matched custom drill guide. After both the 3D printed FSU models and 3D-printed custom drill guide were prepared, the TPTD screws will be guided with a 3D-printed custom drill guide and introduced into the 3D printed FSU models. No significant statistical difference in screw trajectory angles was observed between the digital model and the 3D-printed model (*P* > 0.05). Our present study found that, with the help of CAD software, it is feasible to design a TPTD screw custom drill guide that could guide the accurate TPTD screw trajectory on 3D-printed lumbar models.

## Introduction

Bilateral pedicle screw (BPS) fixation was recognized as the “gold standard” surgical technique for spinal arthrodesis (Boos & Webb, 1997; Suk et al., 2012; Umeta & Avanzi, 2011); however, it still had some disadvantages, such as extensive paraspinal dissection and blood loss, significant cost and surgical wound infection (Adogwa et al., 2011; Koutsoumbelis et al., 2011; Rajaee, Kanim & Bae, 2014), which prompted researchers and surgeons to develop alternative, less invasive techniques. One of the alternative techniques developed in recent years was transpedicular transdiscal (TPTD) oblique lumbar fixation, first described by Abdu, Wilber & Emery (1994) and Grob, Humke & Dvorak (1996). Further, Wu et al. (2013) and Aghayev et al. (2014) described the feasibility of TPTD screw fixation for use in non-spondylolisthesis patients, but Wu et al. emphasized that the optimal trajectory of the TPTD screw was more difficult to achieve than with BPS. Birkenmaier et al. (2010) suggested the use of a robotic-assisted navigation system to help introduce the TPTD screw percutaneously.

The three-dimensional (3D) printing technique was developed in recent decades (Hull & Lewis, 1991). It facilitates the production of 3D-printed models from a 3D digital model or other electronic data source (Di Angelo & Di Stefano, 2015; Horn & Harrysson, 2012). There are many different 3D-printing methods, including stereolithography, laser sintering, laser melting, laminated object manufacturing and fused filament fabrication (Haverman et al., 2013; Webb, 2000). Materials with different properties, cost and colors could be chosen by researchers according to their preference and the 3D-printing technique they used (Giordano et al., 1996; Guo, Heuzey & Therriault, 2014; Horn & Harrysson, 2012). If the printed device will be implanted in the human body, surgeons should consider whether the material is biocompatible.

It was reported that with CT DICOM data, the 3D digital model can be reconstructed and used for the printing of accurate 3D models (Wu et al., 2015). Lu et al. (2009a) designed a patient-specific drill guide to guide the cervical pedicle screw placement, and they found that this novel drill guide significantly reduced operative time and radiation exposure. In this study, we designed a novel TPTD screw custom drill guide, and assessed its accuracy in guiding the TPTD screw trajectory in 3D-printed lumbar models.

## Materials and Methods

### 3D reconstruction and trajectory simulation

**Figure 1:**
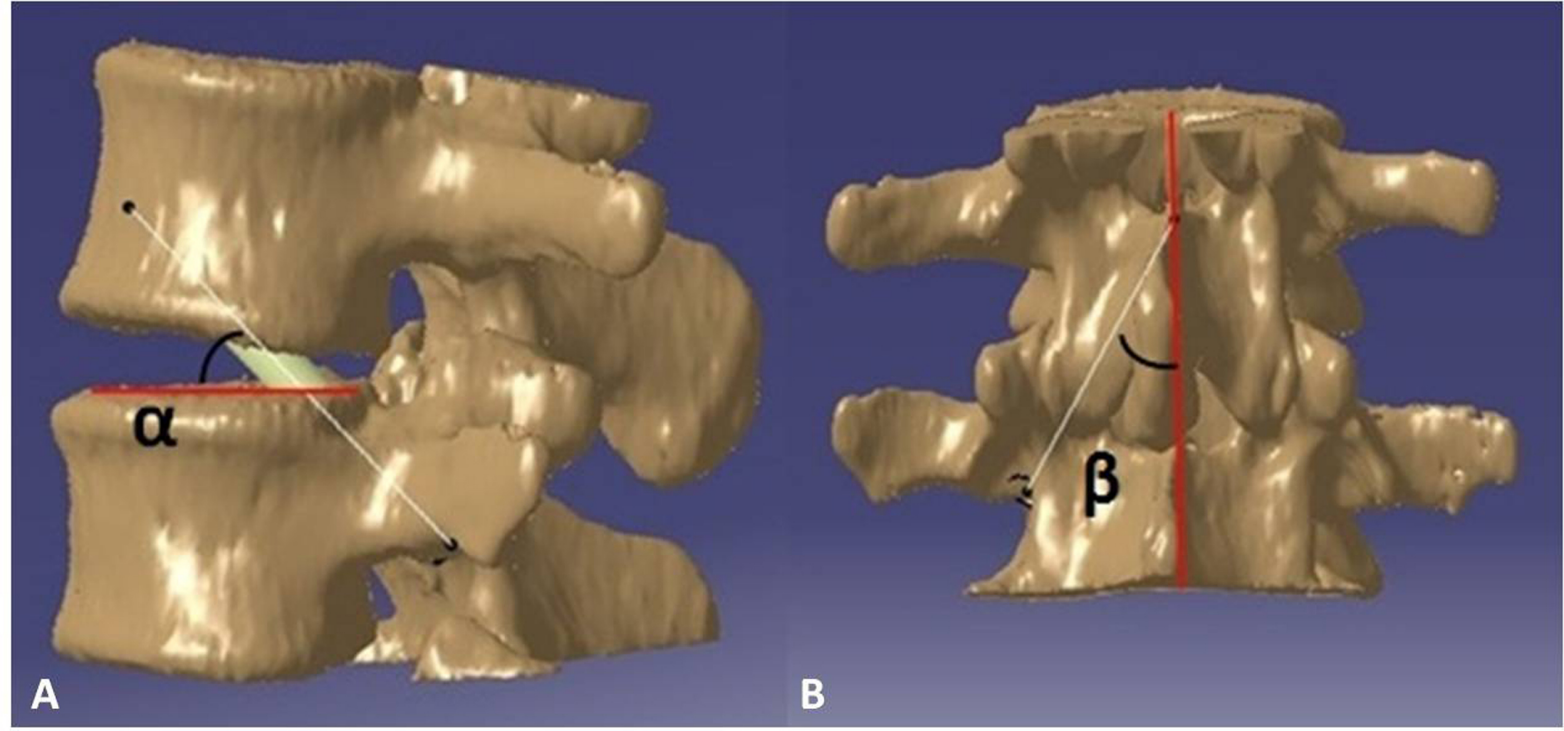
The optimal TPTD screw trajectory was simulated in CAD software, as placed at the central axis of pedicle (latero-median direction) and maximum upward tilt (antero-dorsal direction) while remaining within the pedicle. The angle between the simulated TPTD screw trajectory and the upper endplate of the lower vertebra was measured and termed “α” (A); the angle between the simulated TPTD screw trajectory and the vertical centerline (midline that connects the two adjacent dorsal spinous processes) in an anteroposterior view was measured and termed “β” (B).

Thirty CT DICOM data sets of double lumbar segments (10*L1–2, 10*L2–L3, 10*L3–L4, 120 kv, 150 mA, slice thickness  = 0.45 mm) were exported from the PACS system. The CT data were confirmed to be without any spinal abnormality such as fracture, tumor or spinal deformity. First, the CT DICOM data were imported into the reverse design software for three-dimensional reconstruction, the threshold value was set at “Bone (CT)”, “226-Max”, which is optimal for bone reconstruction; the “Max” value is auto recognized by reverse design software dependent on the bone mineral density of participants. After the 3D digital images were reconstructed, the data were saved in an STL (stereolithography) format. Second, the 3D digital images in STL format were imported into CAD software. As reported in a previous study (Wu et al., 2013), we simulated the TPTD screw trajectory (Diameter: 5.5 mm (L1–L2), and 6.0 mm (L2–L4) (the diameters above are the common screw diameters used for Chinese patients) in CAD software, as placed at the central axis of the pedicle (latero-median direction) with a maximum upward tilt (antero-dorsal direction) while remaining within the pedicle throughout (Fig. 1). The angle between the simulated TPTD screw trajectory and the upper endplate of the lower vertebra was measured and termed “α”; the angle between the simulated TPTD screw trajectory and the vertical centerline (the midline that connect the two adjacent dorsal spinous processes) in the anteroposterior view was measured and termed “β”. Both angles of “α” and “β” were measured with a screen ruler by two independent researchers (ZXS, JSW).

### Custom drill guide design and print

**Figure 2:**
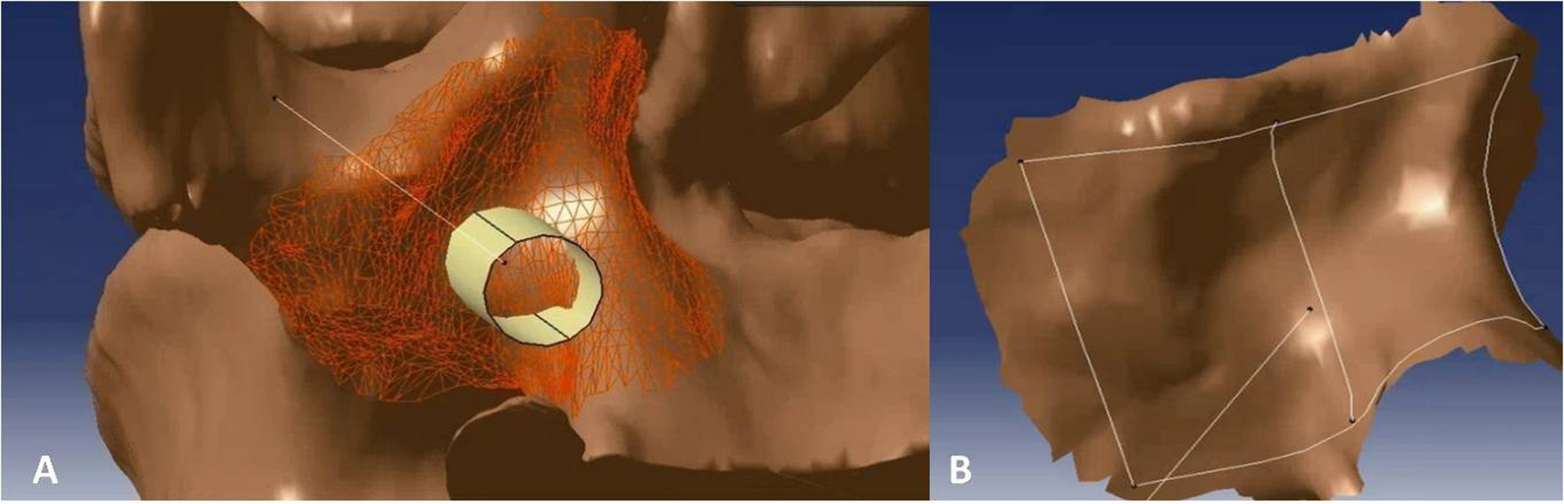
The bony surface (A) around the TPTD screw entry point was extracted to create a near-perfect fitted surface (B).

**Figure 3:**
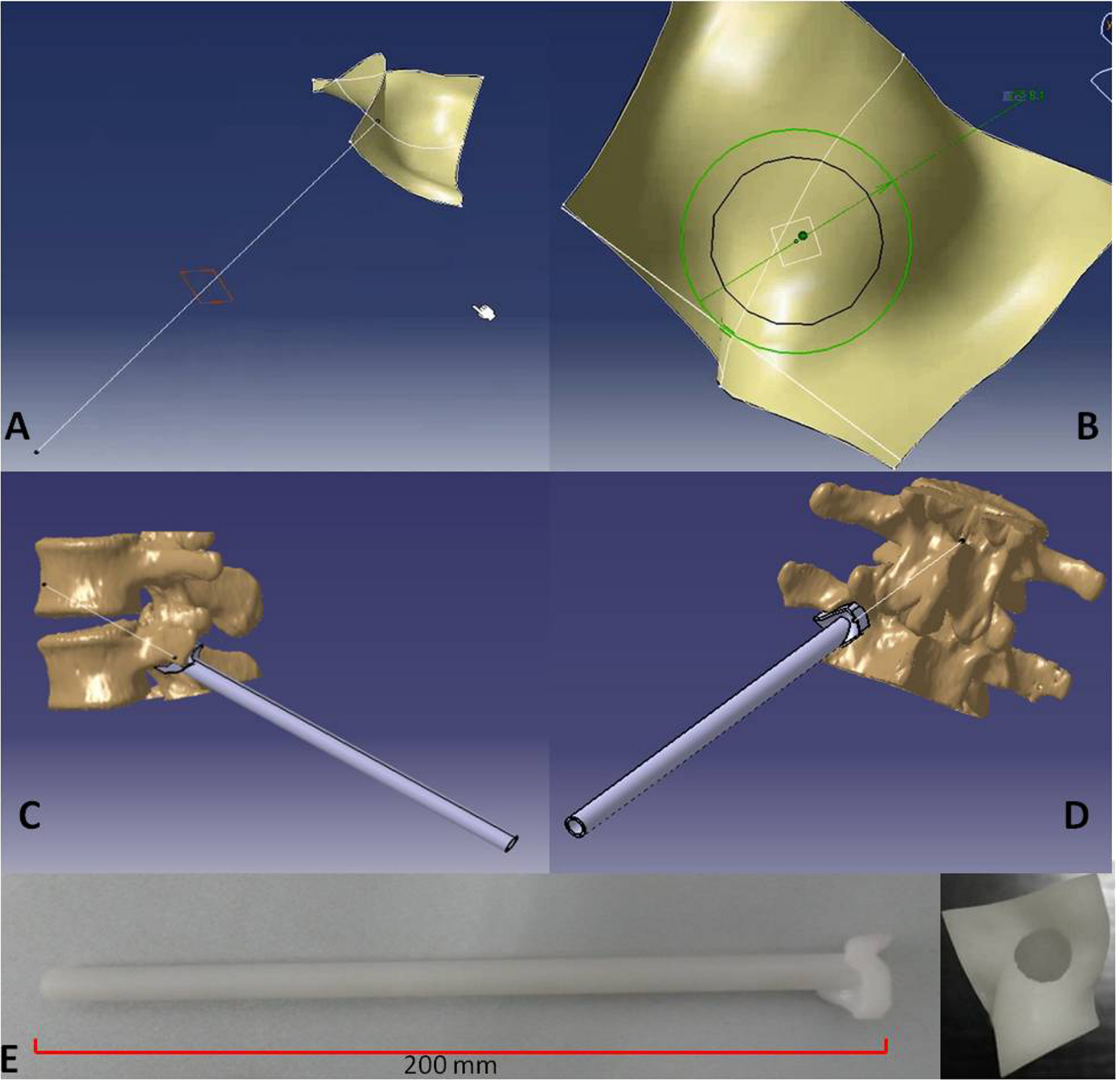
The TPTD screw trajectory was extended to the back forward (A), the hollow “out bone” screw trajectory was created and thickened to approximately 2 mm (B). The fitted surface was thickened to approximately 5 mm, and the 5 mm thick surface and the part of hollow “out bone” screw trajectory was combined as one complete TPTD screw custom drill guide and saved in STL format (C and D). Then, the TPTD screw custom drill guide was sent to the 3D printer to print the plastic TPTD screw custom drill guide (E).

After the optimal TPTD screw trajectory was simulated, the feature of the bony surface around the TPTD screw entry point was extracted, and a new surface with the best fit to the bony surface was created (Fig. 2). The custom drill guide surface was thickened to approximately 5 mm and the TPTD screw trajectory was extended posteriorly 200 mm. The screw trajectory was hollowed-out and widened to a diameter of 2 mm. The 5 mm thick surface and the part of the hollow “out bone” screw trajectory were combined as one complete TPTD screw custom drill guide and saved in STL format (Fig. 3).

The TPTD screw custom drill guide in STL format was imported into the 3D-printing code software, the position was adjusted, saved in Gcode format, and imported into the 3D printer (Meditool Inc., Shanghai, China); curable resin was used to print the plastic TPTD screw custom drill guide (Fig. 3). At the same time, the 3D digital lumbar segments in STL format was converted into Gcode format and imported to a 3D printer (3D ORTHO Waston Med Inc. Changzhou, Jiangsu, China) to print the 3D lumbar modes with PLA (polylactic acid).

### Guide TPTD screw on 3D printed model

**Figure 4:**
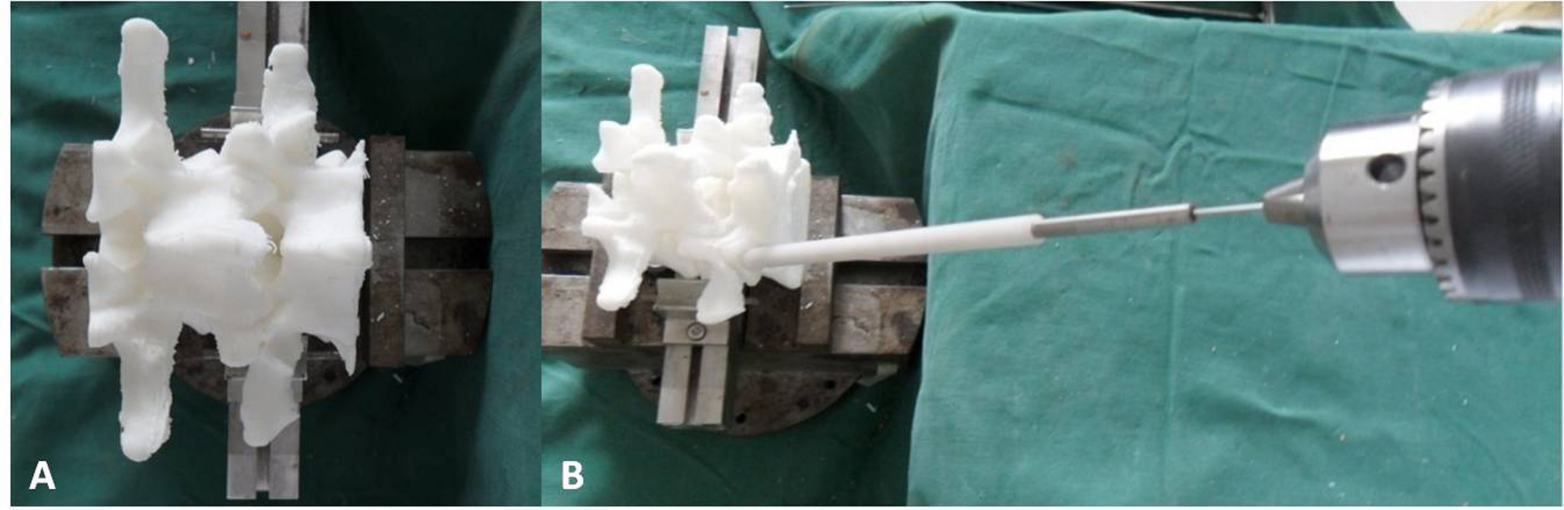
The 3D-printed lumbar model was fixed at the base of support on the table (A). The custom drill guide was placed on the TPTD screw entry point, a guide tube with 5.5 mm or 6.0 mm outer diameter and 1.3 mm inner diameter was inserted inside the hollow custom drill guide. A 1.2 mm diameter K-wire (Kirschner wire) with a sharp tip was placed through the guide tube by power bit drill (B).

The 3D-printed lumbar model was fixed at the base of support on the table, and the TPTD screw custom drill guide was placed on the TPTD screw entry point. To introduce the 1.2 mm diameter K-wire (Kirschner wire), a guide tube with a 5.5 mm or 6.0 mm outer diameter and a 1.3 mm inner diameter was inserted inside the hollow custom drill guide, then a 1.2 mm diameter K-wire with a sharp tip was placed through the guide tube and forward to the intervertebral space and upper vertebral body using a power bit drill (Fig. 4). Once the K-wire was positioned satisfactorily, the guide tube and custom drill guide were removed (the custom drill guide was removed in this study; the surgeon can also preserve the custom drill guide and only remove the guide tube, depending on preference), a screw trajectory was fashioned with a cannulated tap over the K-wire, and the cannulated TPTD screw (with 6 mm/5.5 mm outer diameter and 1.3 mm inner diameter) was introduced over the K-wire. The same procedure was performed on the other side.

**Figure 5:**
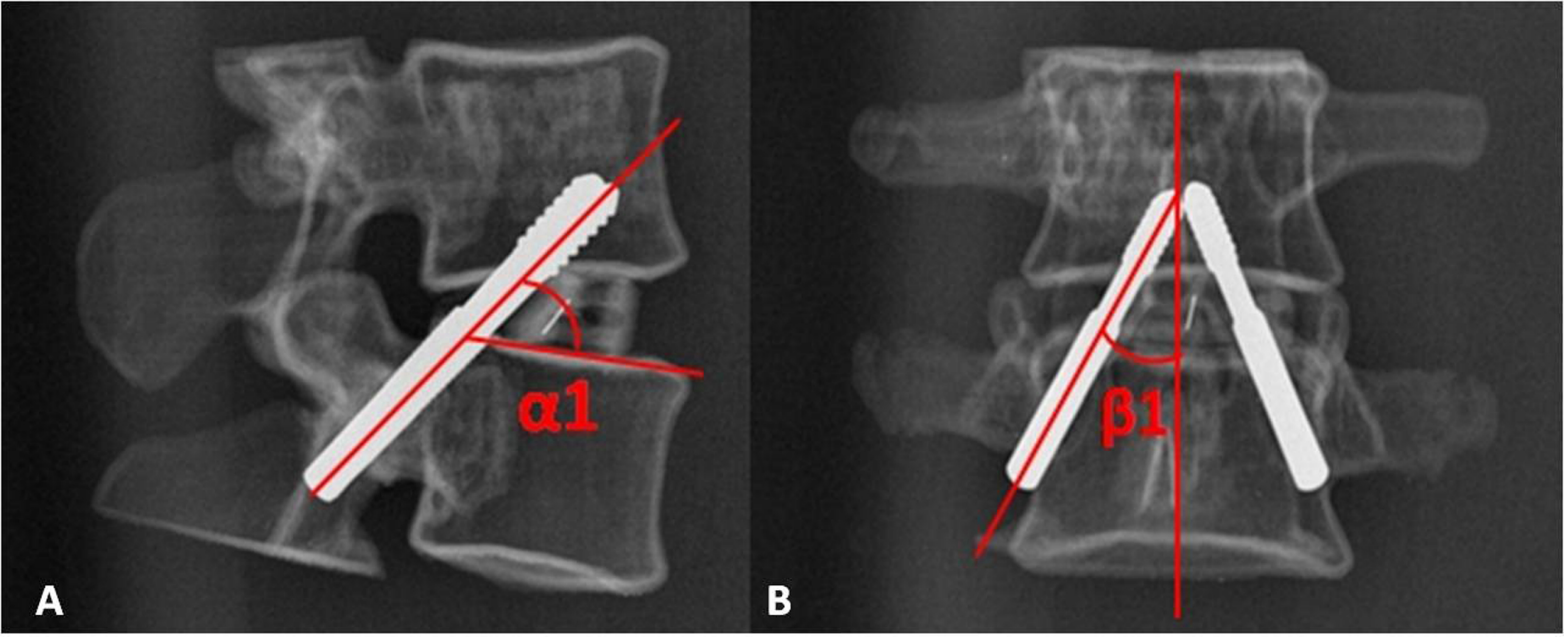
The radiographic lateral and anteroposterior (AP) films of the 3D printed lumbar model were obtained after the TPTD screw was introduced. “α1” is the angle between the TPTD screw and the upper endplate of the lower vertebra measured on radiographic anteroposterior films on the AP films; “β1” is the angle between the TPTD screw and the vertical centerline was measured radiographic lateral films.

The radiographic lateral and anteroposterior (AP) films of the TPTD screw fixed 3D printed lumbar models were obtained at this time (Fig. 5). Four angles were measured: on the lateral films, the angle between the TPTD screw and the upper endplate of the lower vertebra was measured and termed left/right “ α1”; on the AP films, the angle between the TPTD screw and the vertical centerline was measured and termed left/right “ β1”. Both “α1” and “β1” angles were measured with a screen ruler by the same two researchers (ZXS, JSW).

### Ethics consideration

This research was performed in accordance with the principles described in the Declaration of Helsinki and was approved by the Institutional Ethics Review Board of The Second Affiliated Hospital and Yuying Children’s Hospital of Wenzhou Medical University (No. 2015-30). Written informed consent was obtained from all participants.

### Statistical analysis

The data were analyzed with statistical analysis software. The “α” and “β” angles of the simulated trajectory from the CAD software and the “α1” and “β1” angles of the trajectory from radiographic films of the 3D-printed model were compared by paired *t*-tests, with the level of significance set at *P* < 0.05.

## Results

**Figure 6:**
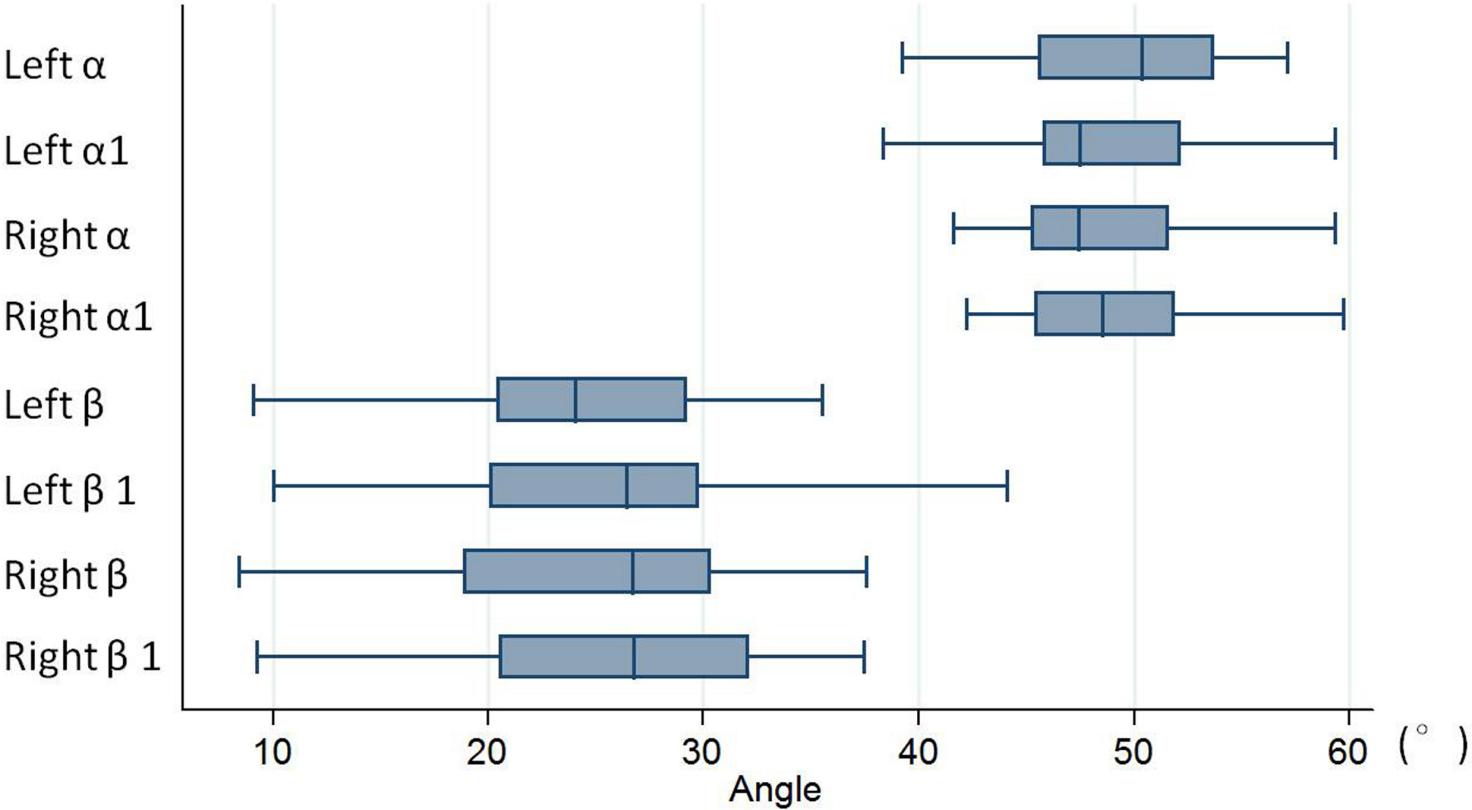
Box and whisker plot of the angle data. No statistically significant difference was observed between the left and right sides (with *P* = 0.138 (Left α *vs.* α1), 0.516 (Right α *vs.* α1), 0.133 (Left β *vs.* β1), 0.674 (Right β *vs.* β1)). “α” is the angle between the simulated TPTD screw trajectory and the upper endplate of the lower vertebra measured on CAD software; “β” is the angle between the simulated TPTD screw trajectory and the vertical centerline (the midline that connect the two adjacent dorsal spinous processes) in the anteroposterior view measured on CAD software; “α1” is the angle between the TPTD screw and the upper endplate of the lower vertebra measured on radiographic anteroposterior films on the AP films; “β1” is the angle between the TPTD screw and the vertical centerline was measured radiographic lateral films. All of above angles are measured both left and right sides.

All TPTD screws were successfully introduced into the 3D-printed model guided by our novel TPTD screw custom drill guide. The angle values of the simulated trajectory in 3D digital images and the screw trajectory in 3D-printed models are shown in Table 1. No statistically significant difference was observed between the left and right sides (*P* = 0.138 (Left α *vs.* α1), 0.516 (Right α *vs.* α1), 0.133 (Left β *vs.* β1), 0.674 (Right β *vs.* β1)). Figure 6 is the box and whisker plot of the angle data.

**Table 1:** Comparisons of “α” and “β” angle of simulated screw trajectory from the CAD software and the “α1” and “β1” angle of screw trajectory from radiographic films of 3D-printed model.

	Angle measured from CAD software	Angle measured from radiographic films	*T* value	*P* value
Left α/α1 angle	49.43 ± 4.84	48.39 ± 4.65	1.525	0.138
Right α/α1 angle	48.46 ± 4.33	48.87 ± 4.51	−0.658	0.516
Left β/β1 angle	24.15 ± 8.47	25.04 ± 7.83	−1.545	0.133
Right β/β1 angle	25.26 ± 7.42	25.43 ± 7.67	−0.425	0.674

## Discussion

Internal screw fixation has been widely used in spinal surgery. Many methods can be used to place an internal screw; some surgeons prefer to place the pedicle screw by free hand, referring to the anatomic landmarks (Kim et al., 2004; Miekisiak et al., 2015; Modi et al., 2008). To improve the accuracy of screw placement, or to place the screw percutaneously, various supporting methods, such as the use of an intra-operative C-arm X-ray monitor, and O-arm monitor and CT, have been studied (Assaker et al., 2001; Chiu et al., 2015; Tjardes et al., 2010). These methods had obvious advantages in the accuracy of screw placement, especially in the context of abnormal spinal structure or for surgeries at the thoracic level (Mason et al., 2014; Tian et al., 2011; Tian & Xu, 2009).

For TPTD screw fixation, the trajectory was different from the transpedicular screw; the entry point was lower, and the screw should be introduced obliquely upward to meet the superior margin of the pedicle where it joins the vertebral body. Therefore, it is more challenging to achieve an optimal TPTD screw (Wu et al., 2013). Birkenmaier et al. (2010) described a study protocol that used the robotic-assisted navigation system to help introduce the TPTD screw. In his protocol, they described that TPTD screw fixation could not only be performed on spondylolisthesis patients but could also be performed on patients with painful disc degeneration (black disc), segmental instability, etc., without spondylolisthesis. In 2013, with the help of a 3D-image guidance system, Nottmeier & Pirris (2013) successfully placed 41 TPTD screws in 12 patients’ thoracic spines. They suggested that TPTD screw fixation had the advantage of purchasing two adjacent vertebral segments across multiple cortical bones, and a high fusion rate was observed across spinal levels in which TPTD screws were placed.

However, the intra-operative radiation exposure associated with C-arm or O-arm use could increase the risk of cancer in surgeons (Mastrangelo et al., 2005; Singer, 2005). Several studies reported the use of a custom drill guide in spine surgery (Berry et al., 2005; Goffin et al., 2001; Owen et al., 2007). Lu et al. (2009a) and Lu et al. (2009b) used a patients’ pre-operative 3D CT data to design the bony surface to fit the custom drill guide to guide the cervical pedicle screw and C2 laminar screw placement. Sugawara et al. (2013) described the use of CT data to design this novel custom drill guide, and used it to successfully guide fifty-eight thoracic pedicle screws. Both Lu et al. (2009a) and Sugawara et al. (2013) suggested that this custom drill guide was simple and could provide an accurate trajectory of the placement of aimed screws while significantly reducing the operating time and radiation exposure.

In this study, the novel TPTD screw custom drill guide was designed based on the CT scan data. Compared to the custom drill guide in studies by Lu et al. (2009a) and Sugawara et al. (2013), this TPTD custom drill guide did not include the bony surface of the laminar. The entry point of the TPTD screw looks like “the human axilla” in region L1–L4 (Wu et al., 2013); the bony surface here is very irregular. A more irregular shape can influence the accurate entry point and screw trajectory. Therefore, the bony surface extracted by this TPTD screw custom drill guide was smaller than that in the report of Lu et al. (2009a). However, the screw trajectory guided by this custom drill guide did not show statistically significant differences compared to the optimal screw trajectory in the CAD software.

The creation of an accurate fitted-surface to bony surface was important to achieve an accurate screw trajectory. The CAD software was used to design the TPTD screw custom drill guide in this study; the “Shape Sculptor” and “Quick Surface Reconstruction” functions could help us extract the aimed bony surface conveniently and accurately. Additionally, the custom drill guide designed in CAD software could be exported in STL format, which is the exact format used for further 3D printing.

### Limitations of this study

There were some limitations of this study. First, this was an *in vitro* study; the lumbar model used in this study was a 3D-printed model, not a cadaveric specimen. 3D-printing technology could provide an accurate morphometric lumbar spinal model (Wu et al., 2015), and the 3D-printed lumbar spine model was less expensive than the cadaveric specimen, and was not affected by the problem of cadaveric shortage, which is present in some developing countries with fewer donations (Halou et al., 2013; Zhang et al., 2008). Second, in this study, only the L1–L4 TPTD screw custom drill guide was designed and investigated (the small area around the screw entry point has enough irregular bony landmarks to design the custom drill guide). Our pre-study found that the bony surface of L4–L5 and L5–S1 TPTD screws entry points did not have enough irregular bony landmarks, and a perfect fit of the custom drill guide surface to bony surface was difficult. Therefore, we did not perform an investigation on the L4–S1 region in this study. Third, the soft tissue around the screw entry point may interfere with the fit between the custom drill guide and the bone, therefore, we suggested that soft tissue should be dissected clearly in a clinical investigation.

## Conclusion

The construction of a TPTD screw custom drill guide was convenient with CAD software. Additionally, the 3D-printed TPTD screw custom drill guide could help us achieve an accurate TPTD screw trajectory at a lower cost and could reduce the radiation exposure for clinical investigations.

##  Supplemental Information

10.7717/peerj.3564/supp-1 Supplemental Information 1 Supplementary data Raw data

## Abbreviations

3D: three-dimensional
TPTD: transpedicular transdiscal
FSU: functional segment Unit
BPS: bilateral pedicle screw

## Additional Information and Declarations

### Data Availability

The following information was supplied regarding data availability: The raw data has been provided as a Supplementary File.
